# Co‐Development of an Evidence‐Based Breastfeeding Support Intervention, Optimised for Delivery in Healthcare Settings, and Adaptations for Mothers With Long‐Term Conditions: The Action for Breastfeeding (A4B) Programme

**DOI:** 10.1111/mcn.70159

**Published:** 2026-02-11

**Authors:** Albert Farre, Anna Gavine, Phyllis Buchanan, Louise Wallace, Fiona Lynn, Joyce Marshall, Shona Shinwell, Sara Cumming, Alison McFadden

**Affiliations:** ^1^ School of Health Sciences University of Dundee Dundee UK; ^2^ The Breastfeeding Network Paisley UK; ^3^ School of Health, Wellbeing and Social Care The Open University Milton Keynes UK; ^4^ School of Nursing and Midwifery Queen's University Belfast Belfast UK; ^5^ Department of Nursing and Midwifery University of Huddersfield Huddersfield UK

**Keywords:** behaviour‐change interventions, breastfeeding, maternal health services, pregnancy, social support, stakeholder participation, systematic review

## Abstract

This intervention development study aimed to work with a wide range of stakeholders across the UK to integrate existing global evidence on the effectiveness and implementation of breastfeeding support for mothers with/without long‐term conditions and co‐develop a complex intervention optimised for delivery in healthcare settings. The intervention development process was informed by four systematic reviews, conducted alongside an embedded programme of co‐production work between 2020 and 2025, involving: two stakeholder working groups (SWG) and two parent panels (PP) that met at regular intervals during the study; six focus group discussions (FGD) to ensure engagement with parents from socially disadvantaged groups; and 10 co‐production workshops (Co‐PW) involving parents, third sector organisations, healthcare practitioners, managers, commissioners, policymakers, and academics. Systematic reviews synthesised data from 116 randomised controlled trials and 16 process evaluations of breastfeeding support interventions for healthy mothers; and 22 trials and 24 studies on views/experiences of breastfeeding support in mothers with long‐term conditions. The co‐production work involved 23 stakeholders and 16 parents in SWG and PP meetings, 15 parents in FGD, and 128 stakeholders in Co‐PW. The resulting Action for Breastfeeding (A4B) Programme comprised four core components (antenatal, postnatal, follow‐up, and signposting) with associated implementation strategies, mechanisms of action, and outcomes for evaluation. Materials and guidance to support adoption and delivery were co‐designed. The A4B Programme provides an evidence‐based and co‐produced intervention to deliver organised support for breastfeeding mothers in healthcare settings, with proposed adaptations for mothers with long‐term conditions. Some uncertainties remain and these will be investigated in our future work.

## Summary

- There is strong evidence that breastfeeding support works, yet many women still stop early due to lack of adequate support, indicating that improvements amply demonstrated in research are not effectively translating into real‐world outcomes.
- This intervention development study combined the use of high‐quality evidence on both effectiveness and implementation, alongside an extensive programme of stakeholder engagement work, to improve potential for context‐adapted real‐world adoption.
- To promote sustainable and inclusive scale‐up, the Action for Breastfeeding Programme is optimised for delivery in healthcare settings and includes adaptations for women with long‐term conditions, ensuring that tailored support can be delivered for all women.

## 1 Introduction

The positive impact of breastfeeding on short, medium and long‐term health outcomes for women and children across the lifespan is well established (Victora et al. 2016). However, in many countries such as the UK, breastfeeding initiation rates are high but there is a rapid decline in continued breastfeeding in the early weeks following birth (Nuffield Trust 2024) and this is when support services have the potential to make a difference. Additionally, few women in the UK breastfeed exclusively for 6 months as recommended by the World Health Organization (WHO).

There is substantial global evidence of the effectiveness of breastfeeding support interventions. Existing high quality evidence suggests that when standalone breastfeeding support interventions are offered to women who choose to breastfeed, the duration and exclusivity of breastfeeding are likely to be increased (Gavine et al. 2022). However, many women in the UK and elsewhere continue to report that a lack of adequate breastfeeding support results in them stopping breastfeeding earlier than planned. It is estimated that around 80% of women in the UK stop breastfeeding before they intended which, in addition to not fully realising the greatest benefits of breastfeeding, causes distress (McAndrew et al. 2012) and can contribute to poor mental health (Rowles et al. 2025). Women report feeling unsupported by healthcare professionals and their social networks, especially in the early weeks following birth (UNICEF UK 2017; Rowles et al. 2025). This is exacerbated by the reduction of breastfeeding support services in many areas. In the UK, research suggests that availability of breastfeeding peer support is variable and not accessed by socially disadvantaged women (Grant et al. 2018) who have the lowest breastfeeding rates. In addition, women may receive inconsistent advice from healthcare professionals who may not value breastfeeding peer support and thus undermine the effectiveness of such interventions (Trickey et al. 2018).

This suggests that population‐wide of breastfeeding support interventions in the UK are failing to transfer the improvements amply demonstrated in research studies (Gavine et al. 2022) to the wider population, particularly among those who need them the most.

To achieve sustainability and spread of evidence‐based interventions, it is crucial to have criteria available for implementers to inform the development of appropriate infrastructure and the adaptation of interventions to different contexts and settings (Skivington et al. 2021). To do so, it is fundamental to have evidence from implementation studies, which can answer questions around the delivery of interventions (e.g. what is implemented and how?), underlying mechanisms of change (e.g. how does the delivered intervention produces a change in outcomes?), and contextual factors (e.g. how does context affect implementation and outcomes?) (Moore et al. 2015).

When considering adaptations for building sustainable, context‑adapted breastfeeding support programmes, it is also vital to consider a comprehensive and inclusive range of support needs. This can help ensure that interventions are able to deliver tailored support for all women, including those living with long‐term conditions (Williams et al. 2019), whose breastfeeding journeys often involve additional challenges and require coordination with medical care (Gavine et al. 2024). The importance of this approach is underscored by the rising prevalence of maternal chronic conditions (Jølving et al. 2016) and consistent evidence that, although initiation rates are comparable, women with health conditions tend to have lower sustained breastfeeding rates than their healthier peers (Scime et al. 2022; Sokou et al. 2023; Šumilo et al. 2012).

The aim of this study was to synthesise global evidence on the effectiveness and implementation of breastfeeding support interventions for all mothers, including those with long‐term conditions, and to work with a wide range of stakeholders to co‐develop a complex breastfeeding support intervention optimised for delivery in healthcare settings, with adaptations for mothers with long‐term conditions, the Action for Breastfeeding (A4B) Programme.

## 2 Methods

The development process of the A4B Programme is reported according to the Guidance for reporting intervention development studies in health research (GUIDED) (Duncan et al. 2020). The intervention development process was informed by four systematic reviews conducted alongside an embedded programme of co‐production over 5 years, between 2020 and 2025. A full report of the reviews and co‐production activities between 2020 and 2023 is published elsewhere (Gavine et al. 2024). The remaining co‐production work (2023–2025) and the intervention development process is originally reported as part of this paper.

### 2.1 Purpose of the Intervention

The primary purpose of the A4B Programme is to support the implementation and delivery of evidence‐based breastfeeding support in National Health Service (NHS) settings focussing on populations where breastfeeding rates are lowest. Whilst the co‐development process was designed to ensure that the resulting intervention was suitable for delivery in NHS settings, it was not designed to be exclusively delivered by NHS services.

### 2.2 Context for Which the Intervention Was Developed

The intervention development process was informed by evidence syntheses of global evidence, but the co‐development work was UK focused, with a view to ensuring that the resulting intervention was suitable for implementation and delivery in NHS settings in Scotland, England, Wales, and Northern Ireland.

### 2.3 Target Population

Healthy breastfeeding mothers with healthy term babies, with planned adaptations to better meet the needs of breastfeeding mothers with long‐term conditions.

### 2.4 Intervention Development Approach

Our intervention development process combined a partnership approach with theory‐ and evidence‐based approaches to intervention development.

### 2.5 Evidence Sources

The main intervention development process was informed by evidence from two systematic reviews; a review of effectiveness and a review of implementation studies, both of which focus on support for healthy breastfeeding mothers with healthy term babies.

The development of adaptations for breastfeeding mothers with long‐term conditions was informed by two further reviews, also including a review of effectiveness and a review of implementation studies, mirroring the evidence base sought for the main intervention development process.

The scope and methods of each review are briefly outlined below:

#### 2.5.1 Evidence Syntheses of Support for Healthy Breastfeeding Mothers With Healthy Term Babies

- Review 1: Update of Cochrane review “Support for healthy breastfeeding mothers with healthy term babies.” The Cochrane Pregnancy and Childbirth's Trials Register was searched in May 2021. Healthy women and babies were those who did not require additional medical care. Interventions could be delivered as standalone breastfeeding support interventions, or as part of a wider maternal and newborn health intervention where additional services are provided (e.g., vaccination, intrapartum care). Primary outcomes were stopping any or exclusive breastfeeding at 6 months and 4‐6 weeks postpartum. Standard Cochrane methods for data extraction, risk of bias assessment, and statistical analysis were used. Meta‐regression was used to investigate statistical heterogeneity (for a full report, see Gavine et al. 2022).
- Review 2: Mixed‐methods review of process evaluations linked to effective breastfeeding support interventions from Review 1. Six electronic databases were searched in March 2022. Eligible studies reported the views and experiences of delivering or receiving breastfeeding support interventions identified as effective in the Cochrane review. Qualitative and quantitative findings were synthesised separately and then integrated into a theoretically‐informed cross‐study synthesis (for a full report, see Gavine et al. 2024).

#### 2.5.2 Evidence Syntheses of Support for Breastfeeding Mothers With Long‐Term Conditions

- Review 3: Effectiveness of breastfeeding support for women with long‐term conditions. Searches were conducted in August 2022. Included studies involved women with a long‐term physical or mental health condition. Primary outcomes were stopping any or exclusive breastfeeding at 4‐8 weeks and 6 months. Standard Cochrane methods for data extraction, risk of bias assessment, and statistical analysis were used (for a full report, see Gavine et al. 2024).
- Review 4: Mixed‐methods review of experiences of breastfeeding support for women with long‐term conditions. Searches were conducted in October 2022. Included studies reported primary research on the views and experiences of breastfeeding women with long‐term conditions and/or support providers. Qualitative and quantitative findings were synthesised separately and then integrated into a theoretically‐informed cross‐study synthesis (for a full report, see Gavine et al. 2024).

### 2.6 Theoretical Framework

The overall framework that informed the design of the A4B Programme was the social ecological model, which understands health as a function of both individuals and the environments in which they live, including family, social networks, organisations, communities and societies (Richard et al. 2011). Therefore, the A4B Programme relies on a series of behaviour change (or, rather, behaviour maintenance) methods to target theoretical variables from multiple levels within an ecological system. The levels of intervention addressed by the A4B Programme and their theoretical underpinnings are as follows:

- Individual level: We aimed to address mothers' perceived ability to breastfeed their babies, which is a salient variable in breastfeeding duration (Dennis 1999). Our chosen methods for change are underpinned by breastfeeding self‐efficacy theory (Dennis 1999) and include performance accomplishments (e.g. past breastfeeding experiences), vicarious experiences (e.g. watching other women breastfeed); verbal persuasion (e.g. encouragement from influential others, such as friends, family, and lactation consultants); and physiological responses (e.g. fatigue, stress, anxiety).
- Interpersonal level: We aimed to address the environmental condition that relationships and social networks provide different types of supportive functions (including emotional, instrumental, informational, companionship, and validation) (Wills and Shinar 2000) which are theoretically associated with a range of health protective effects, particularly under stressful conditions (Eckenrode and Hamilton 2000). Our chosen methods for change at this level are underpinned by social support theory (Eckenrode and Hamilton 2000) and include enhancement of existing networks, use of lay health workers, and linking to new networks.
- Organisational level: We aimed to address organisational processes that can influence the adoption, implementation and continuation of the A4B Programme. Our chosen methods for change at this level are underpinned by the Consolidated Framework for Implementation Research (CFIR) (Damschroder et al. 2022) and include organisation‐ and team‐level implementation strategies.

### 2.7 Components From Existing Interventions

The initial core components of the A4B Programme structure were to be informed by effectiveness data from Reviews 1 and 3, however, Review 3 did not identify any effective interventions for women with long‐term conditions. Therefore, our initial prototype intervention was based on effectiveness data from Review 1, which included 116 randomised controlled studies from 42 countries, involving 98,816 healthy mother‐infant pairs (Gavine et al. 2022). This review suggested that: (1) providing women with extra organised support helps them breastfeed their babies for longer; (2) breastfeeding support may be more helpful if it has 4–8 scheduled visits over the antenatal and postnatal period; and (3) different kinds of support may be needed to meet the needs of diverse groups in specific locations.

From this evidence base, an initial prototype intervention was co‐designed based on a selection of interventions (Bonuck et al. 2014; Cavalcanti et al. 2019; Fu et al. 2014; Hoddinott et al. 2012; McLachlan et al. 2016; Unger et al. 2018; Wu et al. 2020) which met the following two criteria: (1) demonstrated statistically significant reductions in the number of women stopping breastfeeding; and (2) reported evidence at low risk of bias, using allocation concealment as a proxy for this. The prototype intervention was then adapted, refined and optimised through co‐production work (see Section 2.9 below) to ensure its suitability for delivery in NHS settings, until we arrived at the final structure of the A4B Programme reported in Section 3.1 below.

### 2.8 Guiding Principles During the Intervention Development Process

To ensure joint ownership (National Institute for Health and Care Research 2024) our overall approach to stakeholder engagement was “active involvement,” defined as “the contribution of any person who would be a knowledge user but whose primary role is not research” throughout the process of evidence synthesis (including planning, production and dissemination) (Pollock et al. 2018) and intervention development.

### 2.9 Stakeholder Engagement

Stakeholders and parents were involved through two Stakeholder Working Groups (SWG) and two Parent Panels (PP), which met at regular intervals during the study, followed by a series of co‐production workshops.

#### 2.9.1 Stakeholder Working Groups and Parents Panels

One SWG (*n* = 11) and one PP (*n* = 9) focused on healthy breastfeeding mothers with healthy term babies, and the other SWG (*n* = 12) and PP (*n* = 7) focused on breastfeeding mothers with long‐term conditions. SWG members included representatives from: third sector organisations; health professionals; breastfeeding support workers; community breastfeeding support services; national infant feeding networks; and national policy. PP members comprised parents with recent and varied breastfeeding experiences; and for the long‐term conditions panel, parents also had lived experience of physical and mental health conditions (including diabetes, lupus, fibromyalgia, inflammatory bowel disease, multiple sclerosis, hypertension, kidney disease, connective tissue disorders, asthma, chronic fatigue syndrome, anxiety and depression).

Recruitment of SGW and PP members was undertaken via the researchers' professional networks and third sector organisations from across the four UK nations (including Breastfeeding Network, Association of Breastfeeding Mothers, La Leche League, the National Childbirth Trust, Lactation Consultants of Great Britain, and the British HIV Association) and via third sector organisations' Facebook groups.

All SWG and PP meetings were held online between 2020 and 2023.

Alongside PP meetings, we undertook additional focus group discussions (FGD) to ensure engagement with parents from populations less likely to participate in research and/or less likely to breastfeed. FGD participants were recruited via a not‐for‐profit organisation providing general parenting peer support for families living in economically deprived, ethnically diverse populations in England. All FGD participants were offered the choice to attend online or in‐person sessions, with total of 15 participants taking part in one or more FGD, including: eight participants attending the first FGD (five online and three in‐person), six participants attending the second FGD (three online and three in‐person), and nine participants attending the third FGD (six online and three in‐person).

The sequence of engagement activities throughout the project and the main focus and outcomes of activities were as follows:

1. Healthy mothers – First round of engagement activities: The first round of meetings started with activities for participants to get to know each other and agreement of ground rules. The study team then presented the project and provided brief training on systematic reviews. Following this, in their respective meetings, SWG members undertook breakout discussions on how to assess the transferability of breastfeeding interventions to the UK, and PP members and FGD participants reflected on their personal experiences of breastfeeding support and offered their views on important components of support (including who, where, when and how).
2. Healthy mothers – Second round of engagement activities: In their second meeting, SWG members undertook an interactive exercise to score and rank transferability criteria developed by the research team, drawing on the outcome of SWG discussions in round 1 and the PIET‐T model (Schloemer and Schröder‐Bäck 2018). PP and FGD participants were presented with visual materials in plain language covering the transferability criteria ranked by SWG members, and discussed important factors to take into account when transferring interventions from another country to a UK setting.
3. Mothers with long‐term conditions – First round of engagement activities: The first SWG and PP meetings for mothers with long‐term conditions mirrored the same format of the first SWG and PP meetings for healthy mothers. Starting with activities designed for participants to get to know each other and agree ground rules, followed by a presentation of the project from the study team, and a brief training session on systematic reviews. In their respective meetings, SWG members undertook breakout discussions on how to assess the transferability of breastfeeding interventions to the UK and how they supported women with long‐term conditions to breastfeed, and PP members reflected on their personal experiences of breastfeeding support.
4. Healthy mothers – Third round of engagement activities: The third round of engagement activities started with a presentation of a selected set of five diverse interventions identified as effective in Review 1. In their respective meetings, SWG members identified potential implementation barriers and proposed strategies to overcome them, and PP and FGD participants discussed positive and negative aspects for parents, barriers to access and strategies to overcome them.
5. Healthy mothers – Consensus‐building exercise: SWG and PP members were invited to take part in a 2‐stage modified Delphi study (Maxey and Kezar 2016) which presented them with 18 implementation barriers (from previous meetings) and they were asked to recommend implementation strategies that could help address them, from a list of 10 composite strategies adapted from the Expert Recommendations for Implementing Change (ERIC) framework (Powell et al. 2015). For each barrier, strategies with > 70% consensus were taken forward to Stage 2, where respondents were asked to rank in order of importance individual strategies from the themes that reached consensus in Stage 1.
6. Mothers with long‐term conditions – Second round of engagement activities: SWG and PP meetings were presented with a selection of interventions identified as effective for healthy mothers in the main study and discussed them in relation to the needs of women with long‐term conditions and proposed adaptations where needed.

#### 2.9.2 Co‐Production Workshops

Following these engagement activities, the research team developed a prototype intervention which was then further developed and refined through a series of co‐production workshops with representatives from third sector organisations, parents, healthcare practitioners, service managers and commissioners, policymakers, and academics. We undertook a total of 10 co‐production workshops, organised in two phases:

- Phase 1 (2022‐2023): We organised four 1‐day, in‐person workshops across the UK (i.e. one each in Scotland, England, Wales and Northern Ireland). Workshops participants (*n* = 87) were recruited via professional networks of the research team and SWG members, with a focus on participants who represented or worked with communities where breastfeeding rates are low, to maintain the focus on inequalities. Participants worked in small groups of six to eight people on four hands‐on activities focusing on: (1) Adapting the prototype intervention for women with long‐term conditions; (2) Identifying barriers to implementation of the prototype intervention for healthy women and women with multi‐morbidities; (3) Prioritising strategies to overcome implementation barriers identified; (4) Considerations for evaluating breastfeeding support interventions. Throughout all activities, participants were asked to focus on women from communities with low breastfeeding rates. Outputs from all workshop activities and researchers/facilitators notes were collated and analysed using a summative approach to qualitative content analysis (Hsieh and Shannon 2005), involving an initial stage of pattern analysis (i.e. frequency of emerging suggestions within small groups for each of the four activities) followed by an interpretive stage, where the research team met to bring together their notes and insights from their facilitation to contextualise the emerging data patterns and compare/categorise activity‐level patters across small groups and workshop sessions.
- Phase 2 (2024–2025): To continue refining our intervention and get specific feedback on its feasibility and any further adaptations needed for delivery in NHS settings, we undertook three in‐person co‐production workshops with a local NHS Scotland Infant Feeding team, serving a population with high levels of deprivation and low breastfeeding rates. The resulting work was taken to a Scotland‐wide co‐production workshop, attended by participants (*n* = 26) from NHS teams, parents, third sector organisations, and policymakers. This was followed by a further co‐production workshop with the NHS Tayside Infant Feeding team to discuss and integrate refinements. After each workshop, outputs from all activities were collated alongside researchers/facilitators notes and analysed using a summative approach to qualitative content analysis (Hsieh and Shannon 2005), according to the same procedure employed in Phase 1. Following this, we engaged with a graphic designer to develop user‐friendly intervention materials. Finally, we convened an online workshop to consider acceptability and accessibility of final content and visual appearance. The online workshop participants (*n* = 15) included representatives from the NHS and third sector organisations from Scotland, England, Wales and Northern Ireland. This was followed by six iterations of content revision and graphic design changes.

### 2.10 Ethical Statement

Ethics approval was secured through the University of Dundee's Research Ethics Committee (UOD‐SHS‐2021‐010).

## 3 Results

The Action for Breastfeeding (A4B) Programme is described below, in line with the Template for Intervention Description and Replication for Population Health and Policy interventions (TIDieR‐PHP).

### 3.1 Intervention Components, Intensity and Mode of Delivery

Based on the co‐designed prototype intervention informed by the effectiveness data from Review 1 and through multiple iterations of co‐production work to adapt, refine and optimise the prototype intervention to fit the UK context, the final structure of the A4B Programme comprised the four core components outlined in Table 1.

**Table 1:** A4B Programme structure.

Components	Intensity and timepoints	Mode of delivery	Settings/locations	Focus of support sessions
1. Antenatal	One individual appointment, at least 30 min long.	In person.	Healthcare setting or at home, according to preference or convenience.	Focus of antenatal session will involve rapport building, education, and identifying any concerns regarding breastfeeding.
2. Postnatal	One individual appointment, at least 30 min long, before discharge from hospital/midwife‐led unit, or at a similar timepoint for homebirths.	In person.	Healthcare setting (e.g. hospital or midwife‐led unit) except for homebirths.	Focus of postnatal visits will involve checking latch, help with positioning and offering to observe a feed. Breastfeeding support workers will also provide encouragement, information and reassurance during visits. Women will be given the chance to ask questions and raise any concerns.
	One individual appointment, at least 30 min long, within 48 h of discharge from hospital/midwife‐led unit, or at a similar timepoint for homebirths.		Home.	
3. Follow‐up	Month 1: Weekly proactive phone call. Women can contact breastfeeding support workers as needed via phone or SMS if new issues arise.	Delivered remotely (via phone), unless in‐person visit required.	No set location if fully remote. If a visit is needed, this may be delivered at home or in a healthcare setting, according to preference or convenience.	Focus of all follow‐up contacts is to offer support and answer any questions.
	Months 2–3: Monthly proactive call. Women to contact breastfeeding support workers as needed via phone or SMS if new issues arise.			
	Month 3 onwards: Reactive follow‐up as needed.			
4. Signposting to peer support	Signposting to be embedded into and delivered as part of the other components.	Delivered either in person or remotely.	Healthcare setting or at home when embedded into antenatal or postnatal contacts. No set location when delivered with remote follow‐up contacts.	Focus of signposting to peer support will be local breastfeeding peer support groups if available, which may in‐person support groups and phone or social media based (e.g. WhatsApp). Signposting to fully remote peer support may be considered if no local groups are available.

### 3.2 Mechanisms of Action, Mediating Variables, and Intended Outcomes

**Figure 1:**
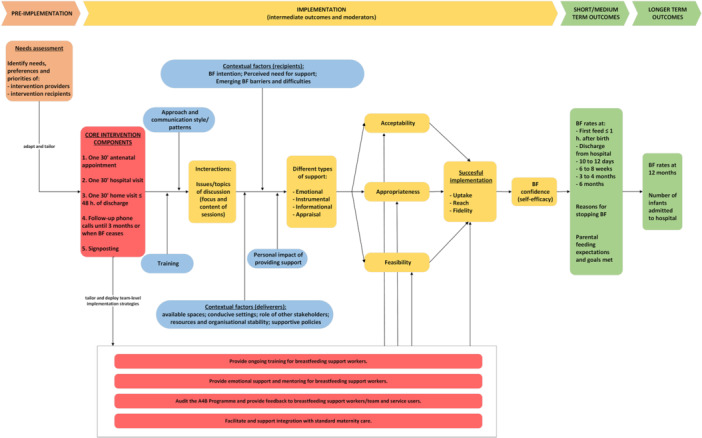
A4B process model.

Based on our analysis of the process evaluation data from Review 2, which included 16 studies (Gavine et al. 2024) and through our multiple iterations of co‐production work, we developed the A4B process model (Figure 1) which attempts to capture how the components A4B Programme can engage mothers, families and providers in supporting breastfeeding. This model attempts to represent the mediating variables related to the A4B Programme which may strengthen engagement with sources of support over time to create breastfeeding behaviour maintenance, ultimately leading to the intended public health outcomes. Identification of determinants was informed by the CFIR framework (Damschroder et al. 2022), and intermediate outcomes were mapped to the Implementation Outcomes Framework (Proctor et al. 2011).

### 3.3 Suggested Implementation Strategies

To support the mechanisms of action outlined above, a range of organisational and team‐level implementation strategies is recommended. Table 2 outlines suggested implementation strategies at organisational level. To facilitate selection and prioritisation by potential adopters, they are mapped against the key barriers they can help address.

**Table 2:** Organisational level implementation strategies.

Implementation strategies	Key barriers addressed by the strategies
Deliver realistic, evidence‐based information in multiple formats on how to deliver the A4B programme and why it is important.	Lack of staff training, knowledge and skills. Lack of consistency of information. Lack of continuity of care. Challenges to accessing the intervention for women and families. Lack of buy‐in from senior managers.
Assign a key practitioner to raise awareness about the A4B programme to ensure a consistent message.	Challenges to working with sectors outside the health system. Poor communication across the multi‐disciplinary team. Lack of joined‐up vision and working.
New or existing funding for breastfeeding support should be a general health investment for local councils and the government, and not just the NHS.	Lack of funding within the health system. Cost of the service to the NHS. Lack of relationship between the health system and the community. Lack of sustainability. Reliance on non‐paid peer supporters.
Create an Infant Feeding or Breastfeeding Support Team in every NHS organisation to lead the A4B programme, working collaboratively with multidisciplinary practitioners and lay supporters.	Lack of availability of good quality training. Time and capacity issues. Professional boundaries – especially working with peer supporters. Lack of confidence of those delivering the intervention. Lack of integration across the continuum (antenatal/postnatal) and across the multi‐disciplinary team.
Revise roles as needed to support the A4B programme – e.g. integrate peer supporters within NHS infant feeding teams, and consider upskilling maternity staff to specialist lactation training levels.	Barriers to integrating peer support with health services including lack of valuing peer support. Lack of right skills mix. Lack of knowledge and skills of staff delivering the intervention. Infant feeding specialists overloaded.

Table 3 outlines the suggested team level implementation strategies, which can help address emerging needs of breastfeeding support workers and service users and help ensure the sustainability of the A4B programme. To facilitate their adoption by local teams, they are presented alongside some practical suggestions to guide further development and tailoring to specific needs of local teams.

**Table 3:** Team level implementation strategies.

Suggested implementation strategies	Practical suggestions for developing and tailoring of implementation strategies to suit local teams' needs
Provide ongoing training for breastfeeding support workers.	Consider organisation and delivery of training to suit a range of work patterns (e.g. part‐time). Delivery format should be engaging and proactive, with a combination of in‐person and online work. Formal training sessions should be delivered at least annually. Follow‐up work may involve small group reflective practice sessions to encourage peer interaction and ongoing informal learning; content should be responsive to needs, helpful for practice, matched to UNICEF UK Baby Friendly Initiative standards and include training on how to involve partners. Facilitate evidence‐based training of trainers and tutors to further develop learning environment in the organisation/team.
Provide emotional support and mentoring for breastfeeding support workers.	Identify and support individual professional development needs, which may include: communication and listening skills, recognising safeguarding concerns, lone worker safety, infant feeding updates, data collection and quality improvement skills, identifying and referring for birth trauma or perinatal mental health support and working with people with learning disabilities. Plan for a range of mentoring and supervision activities, which may include: challenging case reviews/discussions, team meetings, monthly information/updates on topical issues, buddy debrief sessions. Actively support good work‐life balance practices and preservation of boundaries (e.g. scheduling and blocking time for holiday entitlement, being able to switch off work phone and emails when not working).
Audit the A4B Programme and provide feedback to breastfeeding support workers/team and service users.	Consider the ethics (e.g. anonymity, confidentiality, consent) of all elements of feedback collected and all uses the team may want to make of feedback data (e.g. use for training, sharing reports, quality improvement projects). Consider any technical requirements involved in the collection of feedback data (e.g. digital or paper‐based) and how often to share report summaries with staff and service users (e.g. newsletters, social media). Consider any technical requirements involved in the management and storage of feedback data, as well as any access policies required. Consider using feedback to inform current practice (e.g. in ongoing training) and provide support (e.g. resources or access to training) for quality improvement initiatives. Consider collecting different types of feedback data (e.g. qualitative and quantitative) including feedback from staff.
Facilitate and support integration with standard maternity care.	Develop and agree a joint approach with the local maternity team and health visiting team on how to work together to provide breastfeeding support, which may include: establishing who does what (e.g. roles, boundaries and responsibilities), identifying integration champions (e.g. named link or contact person in each team involved), agreeing how to communicate and coordinate across teams (e.g. standardised notes in maternity records), identifying how and when to review joint working arrangements. Identify and address feasibility issues of joint approach arrangements (e.g. tailor coordination efforts to resources available, avoid increasing workload of staff providing standard care, ensure the required buy‐in from management is in place, ensure the intervention has protected time and roles, agree co‐location arrangements of intervention staff and routine staff teams where needed). Plan for regular formal communication across teams, which may include regular newsletters, joint team meetings allowing time to develop relationships across teams (e.g. share concerns and goals), provision of feedback from staff and service users. Consider organising joint learning events for everyone who provides breastfeeding support (e.g. breastfeeding support workers, infant feeding advisors, maternity care staff, health visitors) and consider involving third sector organisations operating in the area (e.g. peer supporters).

### 3.4 Suggested Materials to Support Intervention Delivery

The A4B Programme is accompanied by a toolkit (Farre et al. 2025) which outlines the intervention components and provides recommendations for adaptation, implementation, delivery and evaluation. Suggested materials to support the delivery of the A4B Programme are outlined in Table 4.

**Table 4:** Suggested materials to support intervention delivery.

Intervention components	Conversation topic guide	Materials	Resources	Other considerations
1. Antenatal	Create a flowchart of conversation content that can be individualised. Conversation content topics should cover what to expect in the early days, normal newborn feeding behaviour, skin‐to‐skin, hand expressing, position and attachment, role of partners/family in supporting breastfeeding and signposting to local support groups.	Utilise props and visual aids, including but not limited to, dolls and knitted breasts and allow parents to practice breastfeeding positions.	Build on existing local, national and international resources. Signpost to global health media links.	Consider monitoring what other topics/questions families bring up, so the conversation content can be updated if something is missing. Ensure families have team contact details if they have further questions.
2. Postnatal	Conversation content recaps antenatal conversation (normal newborn feeding behaviour, skin to skin, hand expressing, position and attachment etc) but also builds on this and includes: signs of effective milk transfer, newborn tummy size, baby wearing, accessing breastfeeding support in community. Conversation content is structured but flexible to individual knowledge base.	Visual aids and props such as dolls, knitted breasts, tummy size aids etc.	Signpost to existing local, national and international resources such as global health media. Build on UNICEF‐UK BFI postnatal conversations. Give information about local breastfeeding support groups in the community.	Review individual training needs for those delivering the intervention. Consider training on safeguarding concerns and lone worker support if they are visiting homes alone. Ensure good communication between breastfeeding support workers and other healthcare professionals (e.g. midwife, health visitor etc).
3. Follow‐up	Create conversation content templates to track what was previously discussed during support calls. Include discussion around recognised milestones or transition periods and discuss how to navigate these and what support might be needed.		Consider how to deliver links to resources via text/email. Consider any resources in non‐digital formats or languages for families at risk of digital exclusion or with language barriers.	Consider training needs for assessing and advising breastfeeding support over the phone.
4. Signposting to peer support	Include prompts to check if they have accessed community breastfeeding support groups.	Consider developing support sheets for families about breastfeeding support in their local area, including national helplines. Develop plans for updating and maintaining this information.		Ensure information is shared at point of discharge from A4B programme to relevant health care professional.

### 3.5 Intervention Providers

The evidence base identified in Review 1 suggested that no significant differences are to be expected in terms of effectiveness associated with who provides the support (i.e. professional or non‐professional) (Gavine et al. 2022). However, it is important that providers are specifically trained to deliver breastfeeding support. Training should be matched to UNICEF UK Baby Friendly Initiative (BFI) standards (UNICEF UK 2025). Breastfeeding support workers may have a health or social care background or may be a lay person with relevant training and experience.

### 3.6 Intervention Changes During the Development Process

The intervention development process was iterative, and progress was incremental as the co‐production work evolved. The core structure of four intervention components, which was initially derived from Review 1 effectiveness data, including the proposed intensity and timepoints, did not significantly change throughout the intervention development process, with the exception of two minor adjustments: (1) appointment length changed to “at least 30 min long” rather than the more prescriptive “30 min long” appointments initially proposed; and (2) location for postnatal timepoints amended to include an explicit reference to homebirth settings. Modes of delivery were not initially pre‐specified for all components, but in the two phases of co‐production workshops the consensus among stakeholders was to establish that antenatal and postnatal components should be delivered in person. The main target focus of support sessions did not change, but the more detailed guidance and suggested materials for content and delivery of sessions were not part of the initial prototype intervention and were introduced and developed in our phase 2 co‐production workshops.

### 3.7 Adaptations for Breastfeeding Women With Long‐Term Conditions

The evidence base identified through Review 3 was relatively limited (including 22 studies, involving 5048 mother‐infant pairs) and suggested that existing interventions had little to no effect on breastfeeding outcomes (Gavine et al. 2024). However, through Review 4, which included 24 studies reporting the views and experiences of breastfeeding support for women with long‐term conditions, we were able to consider a richer body of evidence. Based on the evidence base identified via reviews 3 and 4 and through our multiple iterations of co‐production work, we developed four adaptations to the A4B programme to better meet the needs of breastfeeding women living with long‐term conditions:

1. Longer antenatal appointment. The antenatal appointment should be longer than 30 min. Either one long appointment or several shorter appointments need to be considered. Appointments could be delivered earlier in pregnancy due to the increased chance of preterm birth.
*Rationale:* Women with long‐term conditions will need longer appointments so information can be adapted to their individual situation/condition. The appointment may also need to include compatibility of medicines with breastfeeding and practical support to adapt breastfeeding positions to manage pain and fatigue.
2. Continuity of support provider. Women with long term conditions need continuity with the same person delivering the intervention antenatally and postnatally.
*Rationale:* Women with long‐term conditions have complex situations that require tailored breastfeeding support. Continuity ensures they are not repeating their complex stories at each breastfeeding support interaction and not being provided with conflicting information.
3. Integration with medical care. Breastfeeding support workers should be included in joint obstetric and medical clinics.
*Rationale:* Breastfeeding support needs to be situated within joint clinics to ensure individualised advice on medication and breastfeeding and should consider including a pharmacist. Joint clinics would also reduce the number of multiple appointments women with long‐term conditions have and minimise their time and travel costs.
4. Training for healthcare professionals. Increase healthcare professionals' general knowledge, skills and training around breastfeeding with long‐term conditions.
*Rationale:* Some healthcare professionals may not value breastfeeding support and may therefore provide inconsistent advice which undermines breastfeeding. Training is needed to increase knowledge of breastfeeding with long‐term conditions in the multi‐disciplinary team, including General Practitioners. Supporting women with long‐term conditions to breastfeed should be included in routine breastfeeding training updates for all maternity staff.

### 3.8 Remaining Uncertainties

While the content of sessions is to be personalised depending on the specific needs of participating breastfeeding mothers, we did not investigate what would be the optimal coverage of fundamental support topics that could be delivered proactively should the participating mother not be able to clearly identify or voice their needs.

We could clearly identify that providers are to be specifically trained to deliver breastfeeding support, and that training should be matched to UNICEF UK BFI as a minimum standard, however, we were not able to establish a purpose‐built training package for this intervention as this was beyond the scope of our study. Therefore, there is some uncertainty as to what the optimal training package in terms of required training materials and duration of training might be. While there are many suitable training options available from several external providers and resources, it is unclear as to whether these would be equally accessible to all providers wishing to implement this intervention.

The mechanisms of action we identified and our supporting theory suggest that a range of types of support will need to be provided (i.e. emotional, instrumental, informational, appraisal) and this again will vary according to the needs of participating mothers, however, it is unclear what the required skill‐set of providers should be to appropriately cater for all support types. These aspects (e.g. counselling skills) may or may not be covered by existing training packages and resources.

Finally, in relation to the signposting component, we acknowledge that the availability, type and quality of organised peer support groups will vary significantly based on the local area where the intervention is delivered, with some in remote rural areas perhaps only able to signpost to virtual peer support groups. This could have implications in terms of equity in access and/or effectiveness outcomes in these populations and/or settings. Potential mitigation strategies may include: a greater focus on one‐to‐one peer support, combined with use of remote but accessible modes of delivery (e.g. telephone support) and/or use of remote asynchronous modes of delivery (e.g. mobile text messaging).

## 4 Discussion

In this paper, we described the development process of the A4B Programme in a transparent and structured manner, according to the GUIDED reporting guidance (Duncan et al. 2020) and in line with the latest UK Medical Research Council (MRC) framework for developing and evaluating complex interventions (Skivington et al. 2021). Such structured reporting can help researchers, potential intervention adopters, practitioners and research funders to understand the context and methods that were used to develop the intervention and enable them to make informed judgements about the quality and relevance of the intervention (Duncan et al. 2020); and, ultimately, this information can help guide their decisions about whether to evaluate or implement the A4B programme within their specific context.

The A4B Programme development process has several strengths. The novel integration of adaptations specifically for women with long‑term conditions within a comprehensive programme is an important strength. Given the increasing prevalence of maternal chronic conditions (Jølving et al. 2016) approaches that combine proven, universal breastfeeding support components with targeted adaptations addressing the unique needs of breastfeeding mothers with long‐term conditions (Williams et al. 2019) can help improve widespread, real‐world adoption of inclusive breastfeeding support interventions. We combined the use of high‐quality published research evidence on effectiveness and implementation of breastfeeding support interventions, with an extensive programme of stakeholder engagement work embedded from the early stages of our project conceptualisation and funding application through to the dissemination of the resulting intervention. The participatory process involved a wide range of stakeholders (including parents with current or recent breastfeeding experience, third sector organisations, healthcare practitioners, service managers and commissioners, policymakers, and academics) over a total period of 5 years. The participatory process was essential to enhance the quality and relevance of the evidence syntheses underpinning the A4B Programme (Pollock et al. 2019; Boote et al. 2012) and to inform how these were reflected in the intervention—for example, the evidence base suggested that there are no significant differences to be expected in terms of effectiveness associated with how the support is provided (i.e. whether this is provided face‐to‐face, by phone, through digital technologies, or using combinations of these) (Gavine et al. 2022) however, the structure of A4B Programme proposes a combination of modes of delivery which reflects what stakeholders and parents believed would be better suited to achieve the target focus of support sessions at each timepoint.

Another key strength of the A4B Programme is that, alongside the core intervention components rooted in effectiveness data, we also systematically considered mechanisms of action and factors leading to successful implementation, including potential for scale‐up in real‐world population‐wide implementations. This approach is recommended to ensure that new complex interventions not only have a better chance of being effective when evaluated, but also a better chance of being adopted widely in the real world (O'Cathain et al. 2019). In the A4B Programme, this work resulted in a series of bundled implementation strategies at organisational and team levels to be considered by adopters/implementers, which were designed to support key mechanisms of action and lead to positive implementation outcomes. The effectiveness of these strategies, however, remains to be tested. This will be an integral part of our future work as we progress to efficacy testing and any further intervention refinement that might result from that. As part of this, we will also aim to test the feasibility and efficacy of the A4B Programme as a package – i.e. while the core components of the A4B Programme were drawn from full effectiveness trials which showed statistically significant effects in favour of the intervention, the components have not been tested as a package. It will also be important for future research to assess implementation costs of the A4B Programme and to examine its cost‐effectiveness more generally, as these are key considerations that can shape scale‐up efforts in practice. Our previous systematic review work explored the health economics of breastfeeding support interventions and found that the cost‐effectiveness was uncertain due to the limited number of studies and lack of good quality evidence (Gavine et al. 2024).

One limitation of the intervention development process is that the first 2 years were conducted during the COVID‐19 pandemic, which delayed and changed some of our original plans. In practice, this mainly limited the opportunities to involve our SWG and PP in face‐to‐face meetings, which were entirely conducted online. Therefore, some of the more interactive activities we had initially planned for our meetings had to be adapted for online use to ensure that collaborative work dynamics were maintained.

Another limitation was that a purpose‐built training package for this intervention was not developed, as this was beyond the scope of our study. The landscape of education for breastfeeding supporters is complex and lacks standardisation and consensus on competencies (Mulcahy et al. 2022); and, in Review 1, we found that the training of breastfeeding supporters was variable across trials and often poorly reported/specified (Gavine et al. 2022); therefore, it was challenging to establish a recommendation beyond matching training to UNICEF UK BFI as a minimum standard.

## 5 Conclusion

The A4B Programme provides an evidence‐based and co‐produced package of core intervention components and associated implementation strategies to deliver organised support for breastfeeding mothers in NHS settings. Some uncertainties remain and these will be investigated in our future work as we progress to efficacy testing and any further intervention refinement that might result from that.

## Author Contributions

A.F., A.G. and A.Mc.F. conceptualised and designed the study. A.G. led reviews 1 and 3; A.F. led reviews 2 and 4; all authors were involved in reviews 1‐4. A.Mc.F., P.B. and J.M. led the public involvement and stakeholder engagement process. A.F. and A.Mc.F. led the co‐production workshops, and P.B., L.W., F.L., J.M., S.S. and S.C. supported facilitation. S.S. and S.C. organised all involvement and engagement events and led on the collection, management and collation of participant contributions. A.F. led the intervention development process. All authors were involved in data analysis and data integration activities. A.F. led the writing of the manuscript. All authors contributed to and critically reviewed all versions of the manuscript and approved the final version of this article.

## Conflicts of Interest

The authors declare no conflicts of interest.

## Data Availability

The data that support the findings of this study are available from the corresponding author upon reasonable request.
